# Atlanta Medical College

**Published:** 1880-02

**Authors:** 


### Editorial.

### ATLANTA MEDICAL COLLEGE.

Commencement exercises of the above institution were had on Wednesday night, 3d of March, in presence of a large assemblage of citizens convened in the Opera House to witness the closing scenes of the collegiate course. The eloquent and highly esteemed pastor of the First Baptist Church, Dr. Gwin, opened the exercises with prayer. The venerable and faithful Vice-President of the Board of Trustees, Col. W. L. Mitchell, of Athens, then proceeded to confer the degree of Doctor of Medicine upon the following applicants, who were reported by Dr. J. T. Johnson, Dean, as having passed satisfactory examination before the faculty, and asked that the degree be conferred upon them:

J. P. Abercrombey, of Ala.; H. W. Blair, Ala.; James Blaisdell, Maine; P. R. Bradford, Ga.; W. J. Bramlett, S. C.; C. C. Brock, Ga.; R. J. Bruce, Ga.; H. L. Burt, Ga.; T. F. Chandler, Ga.; I. N. Devine, Ga.; J. B. Edge, Ga.; J. G. Faircloth, Ga.; L. S. Garner, Ga.; Z. T. Grady, Ala.; P. P. Gray, Ga.; J. M. Greer, N. C.; J. M. Harrison, Ga.; J. W. Hollingsworth, La.; W. R. Hollingsworth, S. C.; J. W. Holton, Ohio; J. A. Huddleston, Ga.; W. A. Huddleston, Ga.; J. A. Hurt, Ala.; A. R. Jarrett, Ga.; J. A. Knight; Ga.; J. C. LeGrande, Ala.; J. B. Liddell, Texas; H. G. Logan, Ark.; J. H. McCarty, Ga.; F. B. McRea, Ga.; J. S. Macon, Ala.; C. C. Maddox, Ga.; D. S. Moore, Ala.; E. B. Moore, Texas; W. E. Moore, Ala.; R. T. Nash, Ga.; C. C. Quillian, Ga., T. W. Redwine, Ga.; J. N. Ridley, Ga.; W. B. Ross, Ala.; T. M. J. Russell, Ga.; J. H. Simpson, Ga.; C. H. Smith, Ga.; O. M. Starr, Ga.; J. E. Stephens, Ga.; A. Strickland, Ga.; J. T. Van Horn, Ga.; C. A. White, Ga.

The following physicians were admitted *ad eundem gradum*:

A. F. Hambright, M.D., of South Carolina, and C. L. Strother, M.D., of Georgia.

Then Col. Mitchell, for the Board of Trustees, distributed fifty diplomas, after having made a short address in Latin to the graduating class.

Dr. J. A. Hurt, one of the graduates, was then introduced, and delivered the valedictory address on the part of the class. This consisted of sound advice to his classmates and a pathetic farewell to the faculty and citizens of Atlanta, for whom he expressed the kindest feelings and highest regard. The large audience listened with attention and pleasure to this very entertaining address.

For the faculty of the college, H. H. Tucker, D.D., responded in an elegant address on "The True Physician," in his usual eloquent style. Hoping to get a copy for our pages, we shall not attempt to give any portion of it. We shall try to obtain both of these excellent productions for publication.

Next in order was the presentation of prizes awarded successful competitors.

To Dr. J. H. McCarty, of Georgia, was awarded a case of surgical instruments as the prize offered by the faculty for the best general examination on all the branches.

The prize of a medical case, offered by J. G. Westmoreland, Professor of Materia Medica and Therapeutics, for the best thesis "On the Action of Remedies," was awarded to Dr. C. C. Maddox, of Georgia.

A transfusion apparatus, offered by W. F. Westmoreland, Professor of Surgery, for the best report of the surgical clinic, was awarded to Dr. J. A. Hurt, of Alabama.

The obstetrical forceps, offered by Prof. Taliaferro, for the best examination on obstetrics and diseases of women, was awarded to Dr. L. S. Garner, of Georgia.

The prize of an ophthalmoscope, offered by Prof. Calhoun for the best report of the eye and ear clinic, was awarded to Dr. J. A. Hurt, of Alabama.

A medical case, offered by Prof. Love, for the best thesis on the nervous and vascular relations of the ovaries and uterus to the general system, was awarded to Dr. J. H. McCarty, of Georgia.

The gold medal, offered by Prof. Logan for the best thesis on the relations of chemistry to medicine, was awarded to Dr. J. W. Holton, of Ohio.

The prizes were presented by H. V. M. Miller, Professor of the Practice of Medicine, preceded by an entertaining and humorous address.

The exercises, altogether, were of unusual interest, the graduating class being larger than at any time since the war, and exhibiting a higher average standard of preparation.

